# Extreme CD8 T Cell Requirements for Anti-Malarial Liver-Stage Immunity following Immunization with Radiation Attenuated Sporozoites

**DOI:** 10.1371/journal.ppat.1000998

**Published:** 2010-07-15

**Authors:** Nathan W. Schmidt, Noah S. Butler, Vladimir P. Badovinac, John T. Harty

**Affiliations:** 1 Department of Microbiology, University of Iowa, Iowa City, Iowa, United States of America; 2 Department of Pathology, University of Iowa, Iowa City, Iowa, United States of America; 3 Interdisciplinary Graduate Program in Immunology, University of Iowa, Iowa City, Iowa, United States of America; Case Western Reserve University, United States of America

## Abstract

Radiation-attenuated *Plasmodium* sporozoites (RAS) are the only vaccine shown to induce sterilizing protection against malaria in both humans and rodents. Importantly, these “whole-parasite” vaccines are currently under evaluation in human clinical trials. Studies with inbred mice reveal that RAS-induced CD8 T cells targeting liver-stage parasites are critical for protection. However, the paucity of defined T cell epitopes for these parasites has precluded precise understanding of the specific characteristics of RAS-induced protective CD8 T cell responses. Thus, it is not known whether quantitative or qualitative differences in RAS-induced CD8 T cell responses underlie the relative resistance or susceptibility of immune inbred mice to sporozoite challenge. Moreover, whether extraordinarily large CD8 T cell responses are generated and required for protection following RAS immunization, as has been described for CD8 T cell responses following single-antigen subunit vaccination, remains unknown. Here, we used surrogate T cell activation markers to identify and track whole-parasite, RAS-vaccine-induced effector and memory CD8 T cell responses. Our data show that the differential susceptibility of RAS-immune inbred mouse strains to *Plasmodium berghei* or *P. yoelii* sporozoite challenge does not result from host- or parasite-specific decreases in the CD8 T cell response. Moreover, the surrogate activation marker approach allowed us for the first time to evaluate CD8 T cell responses and protective immunity following RAS-immunization in outbred hosts. Importantly, we show that compared to a protective subunit vaccine that elicits a CD8 T cell response to a single epitope, diversifying the targeted antigens through whole-parasite RAS immunization only minimally, if at all, reduced the numerical requirements for memory CD8 T cell-mediated protection. Thus, our studies reveal that extremely high frequencies of RAS-induced memory CD8 T cells are required, but may not suffice, for sterilizing anti-*Plasmodial* immunity. These data provide new insights into protective CD8 T cell responses elicited by RAS-immunization in genetically diverse hosts, information with relevance to developing attenuated whole-parasite vaccines.

## Author Summary

*Plasmodium* infections are a global health crisis resulting in ∼300 million cases of malaria each year and ∼1 million deaths. Radiation-attenuated *Plasmodium* sporozoites (RAS) are the only vaccines that induce sterilizing anti-malarial immunity in humans. Importantly, “whole parasite” anti-malarial RAS vaccines are currently under evaluation in clinical trials. In rodents, RAS-induced protection is largely mediated by CD8 T cells. However, the quantitative and qualitative characteristics of RAS-induced protective CD8 T cell responses are unknown. Here, we used surrogate markers of T cell activation to reveal the magnitude and kinetics of *Plasmodium*-specific CD8 T cell responses following RAS-immunization in both inbred and outbred mice. Our data show that, independent of host genetic background, extremely large memory CD8 T cell responses were required, but not always sufficient for sterilizing protection. These data have broad implications for evaluating total T cell responses to attenuated pathogen-vaccines and direct relevance for efforts to translate attenuated whole-*Plasmodium* vaccines to humans.

## Introduction

*Plasmodium* infections are a global health crisis resulting in ∼300 million cases of malaria each year and ∼1 million deaths [1], [2], [3], [4], [5]. At present, there are no effective licensed anti-malarial vaccines. Most vaccines under clinical evaluation are only partially protective and, for unknown reasons, immunity rapidly wanes [6]. Thus, development of an effective malaria vaccine that provides long-term protection remains an important goal to improve global health.

Immunization with radiation-attenuated sporozoites (RAS) is the only documented means to induce sterilizing protection in both humans [7], [8] and rodents [9] and, importantly this approach is under evaluation in clinical trials [10]. Studies with inbred mouse strains reveal a prominent and often essential role for CD8 T cells in RAS-induced protection [11]. However, RAS-immune inbred mice also exhibit substantial differences in resistance to challenge with *Plasmodium berghei (Pb)* or *P. yoelii (Py)* sporozoites, two major models of experimental malaria that are thought to differ in virulence. Despite decades of research, the precise characteristics of protective memory CD8 T responses following RAS-vaccination remain poorly understood. One reason for this relates to the limited number of defined CD8 T cell epitopes derived from rodent species of *Plasmodia*.

BALB/c mice mount H-2K^d^-restricted CD8 T cell responses against single defined circumsporozoite (CS) protein-derived epitopes from either *Pb* or *Py* and these epitopes can be targets of protective CD8 T cells [12], [13]. However, despite evidence that non-CS antigens can also be targets of protective immunity [14], [15], there are few additional *Plasmodium*-specific epitopes identified from antigens other than CS in BALB/c mice, and no identified protective epitopes in H-2^b^ C57BL/6 (B6) mice. Thus, the paucity of epitope information for these parasites has contributed to our incomplete understanding of the specific quantitative and qualitative characteristics of RAS-induced CD8 T cell responses in inbred mice that are relatively easy (BALB/c) or difficult (B6) to protect against *Plasmodium* sporozoite challenge [11]. Moreover, we recently showed that the threshold of memory CD8 T cell responses to the *Pb*-CS epitope (monospecific responses) required for sterilizing immunity against sporozoite challenge was extremely large [16]. Importantly, it is unknown whether a more diverse memory CD8 T cell response generated by whole parasite based RAS vaccination will decrease the threshold number of memory cells required for protection. This issue is of great relevance to translation of the attenuated whole parasite vaccines to humans.

The identification and characterization of infection- or vaccination-induced, antigen-specific CD8 T cell populations has historically required defined antigenic peptide determinants with known MHC restriction. However, specific activation markers can be used to track effector, but not memory, CD8 T cell responses to viral vaccines in humans in the absence of defined antigenic determinants or known MHC-restriction [17]. We recently described an alternative surrogate actiation marker approach, relying on concurrent downregulation of surface CD8α and upregulation of CD11a (α-chain of LFA-1) on effector and memory antigen-specific CD8 T cells responding to bacterial and viral-infections in mice [18]. Herein, we apply this surrogate marker approach to identify and longitudinally track the total CD8 T cell response following RAS-immunization in rodents. This surrogate marker approach allowed us for the first time to evaluate CD8 T cell responses and protective immunity to RAS-immunization in both inbred and outbred hosts. Collectively, our data show that despite broadening the number of antigenic targets through whole-parasite vaccination, extraordinarily large numbers of memory CD8 T cells are required, but not always sufficient, to protect the host against liver-stage *Plasmodium* infection. These data provide fundamentally new insight into protective CD8 T cell responses elicited by RAS-immunization in genetically diverse hosts, information with relevance to developing attenuated whole-parasite vaccines to protect humans.

## Results

### The CD8α^lo^CD11a^hi^ T cell phenotype specifically identifies RAS vaccine-induced effector and memory CD8 T cells

**Figure 1:**
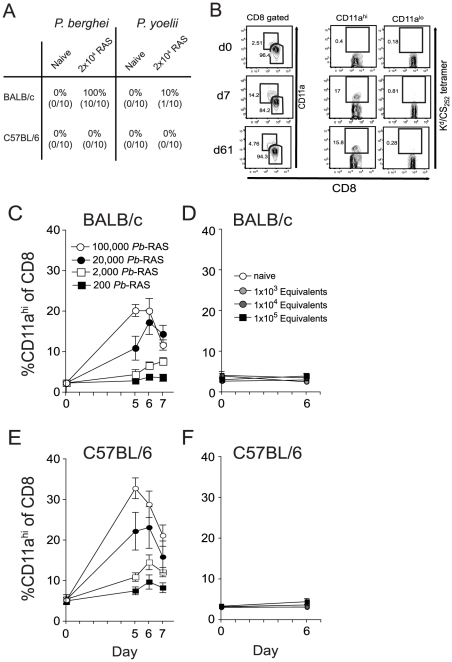
Sensitivity and specificity of the CD8α^lo^CD11a^hi^ surrogate activation marker approach to identify RAS vaccination-induced CD8 T cell responses. (**A**) Protection against *P. berghei* (*Pb*) or *P. yoelii* (*Py*) sporozoite challenge in BALB/c and B6 mice singly vaccinated with either 2×10^4^ *Pb*- or *Py*-RAS and challenged with 1000 *Pb* or *Py* sporozoites >80 days later. Numbers indicate % protected (no. protected/no. challenged×100). (**B**) Peripheral blood mononuclear cells from a single BALB/c mouse collected before (day 0) and after 2×10^4^ *Pb*-RAS vaccination (day 7, 61) were stained for CD8α and CD11a. Left column of dot plots shows the fraction of circulating CD8 T cells exhibiting an antigen-experienced phenotype (CD8α^lo^CD11a^hi^) at each time point following *Pb*-RAS vaccination. Right columns of dot plots show the fraction of cells within the CD11a^hi^ and CD11a^lo^ gates that stain with K^d^/CS_252–260_ tetramer. (**C**) BALB/c mice were vaccinated with the indicated number of *Pb*-RAS and CD8α^lo^CD11a^hi^ responses were tracked in the peripheral blood of individual mice on day 0, 5, 6 and 7. N = 10 mice/dose, except for 1×10^5^ *Pb*-RAS group (N = 5). Data are the mean ± S.D. (**D**) Irradiated salivary gland homogenate from non-infected mosquitoes was injected into naïve BALB/c mice i.v. The CD8α^lo^CD11a^hi^ response was evaluated in the peripheral blood of individual mice before (day 0) and after (day 6) injection. ‘Equivalent’ refers to the final dilution of salivary gland homogenate injected into mice within each group. Dilutions were made based on an average recovery of ∼15,000 sporozoites per mosquito, calculated over >15 independent mosquito dissections. Data are mean ± S.D. for 3 mice per group. (**E**,**F**) similar to **C**,**D** except C57BL/6 mice were analyzed.

Relative resistance after RAS-vaccination of both rodents and humans is commonly studied by sporozoite challenge 1–2 weeks following the last immunization [8], [11], [19], [20] and thus evaluates immunity mediated by recently stimulated T cell populations. Herein, we wished to examine RAS-induced protection only after stable memory immune responses have been generated. Thus, we challenged RAS-vaccinated mice >60 days post-immunization, when numerically and phenotypically stable memory CD8 T cell populations are established following acute infections [21]. At this memory time point, a single *Pb*-RAS vaccination protected 100% of BALB/c mice, but failed to protect any B6 mice against homologous *Pb* sporozoite challenge, whereas one *Py*-RAS vaccination had minimal (BALB/c, 10%) or no (B6, 0%) protective efficacy against homologous *Py* sporozoite challenge (**Figure 1A**). These data demonstrate both mouse strain and *Plasmodium* species-dependent protection after single RAS-immunization of mice challenged at a bona fide memory time point.

To examine the protective CD8 T cell response elicited by RAS-vaccination, we applied our recently described surrogate activation marker approach, based on downregulation of CD8α and upregulation of CD11a (CD8α^lo^CD11a^hi^) [18], to identify RAS-induced CD8 T cells. We chose to focus our initial analyses on peripheral blood (PBL) so that individual mice could be analyzed longitudinally. Importantly, long-term longitudinal analyses of naïve mice in our colony reveal that the circulating CD8α^lo^CD11a^hi^ T cell pool remains low (2–3% of all circulating CD8 T cells) and stable for >250 days (data not shown). For vaccinated mice, the fraction of CD8α^lo^CD11a^hi^ T cells in the PBL was determined prior to, and at various intervals after, immunization with 2×10^4^ *Pb*-RAS in individual animals. We detected substantial increases in the frequency of CD8α^lo^CD11a^hi^ T cells in the blood of vaccinated mice at 7 and 61 days (effector and memory time points, respectively) post-immunization (**Figure 1B** **, left column as an example**). Interestingly, only 16±3% of *Pb*-RAS-induced effector (day 7) CD8 T cells in BALB/c mice are specific for the known H-2K^d^-restricted CS_252–260_ epitope and, importantly, all of these defined antigen-specific CD8 T cells are found in the CD8α^lo^CD11a^hi^ population (**Figure 1B** **, right columns**). Moreover, the fraction of CS_252–260_-specific CD8 T cells among the CD8α^lo^CD11a^hi^ population consistently remains ∼16% throughout the memory phase of the response (day 61) (**Figure 1B** **, right columns**). Based on previous studies showing that T cell responses against diverse epitopes are coordinately regulated [22], these data further support that the surrogate activation marker approach identifies true RAS-induced, *Plasmodium*-specific CD8 T cells. Thus, ∼85% of *Pb*-RAS-induced CD8 T cells in BALB/c mice are reactive against epitopes from undefined antigens. Similar results were obtained for the CS_280–288_ epitope after single immunization of BALB/c mice with *Py*-RAS, although the fraction of CS_280–288_-specific memory CD8 T cells in the circulating CD8α^lo^CD11a^hi^ compartment was only ∼7% (data not shown).

**Figure 2:**
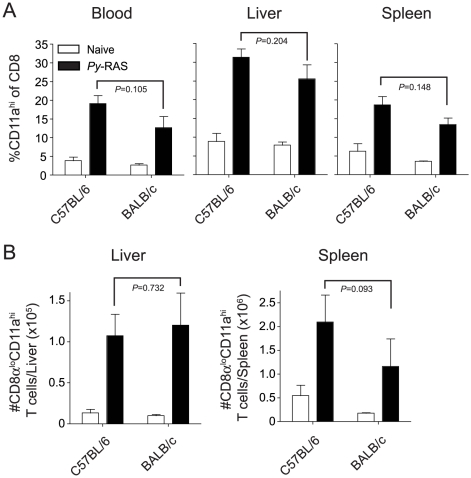
RAS vaccination of BALB/c and C57BL/6 mice increases the frequency and total number of CD8α^lo^CD11a^hi^ cells in spleen, liver and peripheral blood. Naïve BALB/c and C57BL/6 mice were vaccinated with 2×10^4^ *Py*-RAS. Seven days later, mononuclear cells were isolated from the spleen, liver and blood of immune (N = 3/group) and naïve (N = 3) mice and stained for CD8α and CD11a. Data (mean ± S.D.) are expressed as the frequency (**A**) or total number (**B**) of CD8 T cells exhibiting the antigen-experienced phenotype (CD8α^lo^CD11a^hi^) in each tissue. Statistics were determined by unpaired, two-tailed t-tests.

To further demonstrate specificity of the surrogate activation marker approach, we determined that the increase over baseline (PBL analyzed before immunization) in the fraction of circulating CD8α^lo^CD11a^hi^ T cells 5–7 days after immunization depended on the immunizing dose of *Pb*-RAS in both BALB/c and B6 mice (**Figure 1C and E**) and was not observed in mice immunized with an equivalent suspension of irradiated salivary gland homogenates from non-infected mosquitoes (**Figure 1D and F**). Thus, the CD8α^lo^CD11a^hi^ T cell response is specific for *Plasmodium*-antigens and not mosquito salivary gland antigens. Moreover, CD8 T cell responses in the blood of RAS-immune B6 and BALB/c mice were representative of CD8α^lo^CD11a^hi^ responses in the spleen and liver, both in terms of frequency (**Figure 2A**) and total number (**Figure 2B**). Finally, we addressed specificity at the memory stage by transferring sort purified CD8α^hi^CD11a^lo^ (naïve) or CD8α^lo^CD11a^hi^ (memory) cells from day 78 RAS-immune B6 mice (CD45.2) into CD45.1 hosts. Only the population of transferred CD8α^lo^CD11a^hi^ T cells underwent secondary expansion after RAS-immunization of the recipient mice (**Figure S1**). Thus, the CD8α^lo^CD11a^hi^ phenotype cells present at memory time points after RAS-immunization are *Plasmodium*-specific (**Figure S1**). As a composite, these data demonstrate that the changes in frequency of circulating CD8α^lo^CD11a^hi^ T cells in individual RAS-immunized mice reflects the distribution of parasite-specific effector and memory CD8 T cells in peripheral tissues and can be used to evaluate the total CD8 T cell response to RAS-immunization prior to sporozoite challenge.

### Longitudinal analyses of RAS-induced effector and memory CD8 T cell responses

**Figure 3:**
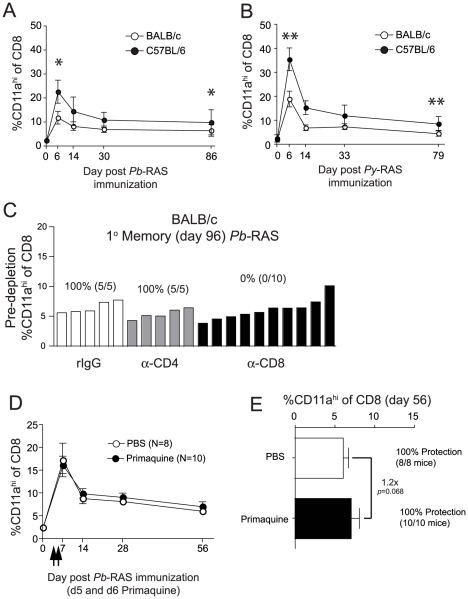
The magnitude and kinetics of RAS-vaccination-induced CD8 T cell responses in BALB/c and C57BL/6 inbred mice. (**A** and **B**) Kinetics and magnitude of the total CD8 T cell response in the blood of BALB/c and C57BL/6 mice following 2×10^4^ *Pb*-RAS (**A**) or *Py*-RAS (**B**) vaccination. Data (mean±S.D.) are from 30 mice/strain. *P<0.0001, **P<0.0001. (**C**) BALB/c mice (N = 20) were vaccinated with 2×10^4^ *Pb*-RAS. Ninety-six days later, the frequency of CD8α^lo^CD11a^hi^ T cells in the peripheral blood was evaluated and individual mice were ranked according to the magnitude of the memory CD8 T cell response. Mice were treated with rat IgG, anti-CD4 (GK1.5) or anti-CD8 (2.43) 3 days and 1 day prior to challenge with 1000 *Pb* sporozoites. Numbers above refer to percent of T cell-depleted mice protected (no. protected/no. challenged ×100) following sporozoite challenge. (**D**) BALB/c mice were vaccinated with 2×10^4^ *Pb*-RAS. On days 5 and 6 post-vaccination, mice were injected i.p. with PBS or 60 mg/kg primaquine/PBS solution (arrows). Circulating CD8 T cell responses were evaluated in mice at the indicated time points. (**E**) Fifty-six days following *Pb*-RAS vaccination, CD8 T cell responses in the blood were evaluated. Mice were challenged 3 days later (day 59) with 1000 *Pb* sporozoites. Numbers refer to % protected (no. protected/no. challenged×100). Data in **D**,**E** are mean±S.D. from 8–10 mice/group. Statistics in **A**, **B** and **E** were determined by unpaired, two-tailed t-tests.

We next examined the magnitude and kinetics of total CD8 T cell responses in the PBL of BALB/c and B6 mice following *Pb*- or *Py*-RAS vaccination (**Figure 3A and B** **, respectively**), using an immunizing dose of RAS (2×10^4^ sporozoites) that fell within the linear range of the CD8 T cell response in both inbred mouse strains (**Figure 1C,E**). We observed substantial increases in the frequency of CD8α^lo^CD11a^hi^ T cells in the PBL of all groups, which peaked 6 days after RAS-immunization, followed by contraction and the formation of numerically stable primary (1°) memory populations (**Figure 3A,B**). Importantly, although B6 mice are more susceptible to sporozoite challenge following a single *Pb*- or *Py*-RAS immunization compared to BALB/c mice [11] (**Figure 1A**), and CD8 T cells are necessary to mediate protection in *Pb*-RAS immune mice (**Figure 3C**), *Pb*- or *Py*-RAS vaccination of B6 mice induced 1° effector and memory CD8 T cell responses that were ∼2-fold higher (*p*<0.0001) than observed in BALB/c mice (**Figure 3A,B**). Thus, our surrogate activation marker approach revealed that the susceptibility of single RAS-immunized B6 mice to homologous *Pb* or *Py* challenge is not due to a diminished total anti-*Plasmodial* CD8 T cell response.

RAS remain infectious to host hepatocytes, but are unable to undergo differentiation into blood stage merozoites [23], [24]. Interestingly, persistence (up to 6 months) of radiation-attenuated parasites was reported in the livers of RAS-vaccinated rats [25] and persistence of attenuated parasites has been hypothesized to underlie the long-term protective capacity of RAS-induced memory CD8 T cells [25], [26]. To address this hypothesis, we treated BALB/c mice with 60 mg/kg primaquine on days 5 and 6 following *Pb*-RAS-vaccination to eliminate persisting parasites. In contrast to previous studies [25], [26], we found that primaquine treatment did not decrease protection against sporozoite challenge at a memory time point (**Figure 3E**). Consistent with this result, primaquine treatment at these time points did not reduce RAS-specific circulating CD8 T cell frequencies (**Figure 3D,E**). In parallel, we verified the efficacy of primaquine (route, dose, schedule) by treating naive BALB/c mice 24 and 48 hrs following challenge with 1000 infectious sporozoites. Primaquine treatment effectively stopped the development of blood stage infection in 100% (5/5) mice, whereas 5/5 vehicle-treated mice developed patent blood stage parasitemia. Thus, following the induction of CD8 T cell responses via *Pb*-RAS-vaccination of BALB/c mice, the persistence of attenuated parasites in the liver does not regulate the stability or protective capacity of the RAS-induced memory CD8 T cell populations.

### Homologous boosting markedly expands RAS-induced memory CD8 T cell populations but fails to confer protective immunity to C57BL/6 mice

**Figure 4:**
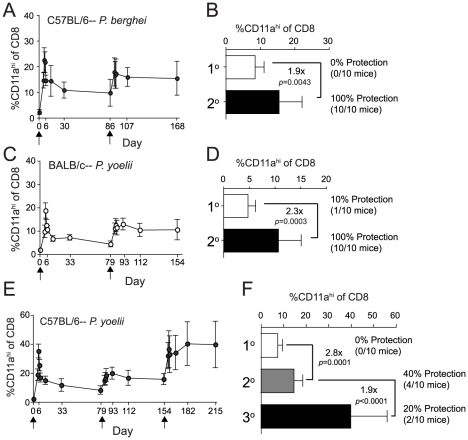
Homologous *Pb*- or *Py*-RAS boosting of mice elicits robust memory CD8 T cell responses but does not enhance protection of C57BL/6 mice against *Py* sporozoite challenge. (**A**) The kinetics and magnitude of the circulating RAS vaccine-induced CD8 T cell response in C57BL/6 mice primed and boosted (arrows) with 2×10^4^ *Pb*-RAS. (**B**) Fold increase in secondary memory (d168) CD8 T cell responses in the blood of C57BL/6 mice following homologous *Pb*-RAS boost. Numbers to the right indicate the fraction of primary (1°) or secondary (2°) memory C57BL/6 mice that were protected following *Pb* sporozoite challenge (no. protected/no. challenged ×100). (**C**,**D**) Similar to **A**,**B**, except BALB/c mice were prime-boosted (arrows) with 2×10^4^ *Py*-RAS and challenged with *P. yoelii* sportozoites. Data in **A–D** are mean±S.D. from 20 mice. (**E**,**F**) Similar to **C**,**D**, except C57BL/6 mice were primed and boosted twice (arrows) with 2×10^4^ *Py*-RAS. Data in **E**,**F** are mean±S.D. from 30 mice. Statistics were determined by unpaired, two-tailed *t*-tests. For **B**,**D** and **F**, one hundred percent (10/10) of strain- and age-matched, naïve mice challenged in parallel were parasitized.

Short-interval (every 2–3 weeks) booster RAS-immunizations improve protection against sporozoite challenge of mice [11], [27], [28] although the impact on the *Plasmodium*-specific CD8 T cell compartment is unknown. Additionally, the impact of long-interval boosting, as generally employed in human vaccines, on RAS-induced protection at a secondary memory time point is unknown. Thus, we examined the effect of homologous RAS-boosting on bona fide memory CD8 T cell populations. Booster immunization at memory time points (60–80 days after initial priming) with *Pb*-RAS in B6 mice or *Py*-RAS in BALB/c mice induced secondary expansion of CD8α^lo^CD11a^hi^ T cells (**Figure 4A,C**). Surprisingly, the peak secondary response did not exceed the magnitude of the peak primary response to initial priming. Still, booster immunization resulted in a doubling of the secondary (2°) memory CD8 T cell populations in both mouse strains (**Figure 4B,D**). Importantly, we observed 100% protection in *Pb*-RAS-vaccinated B6 mice and *Py*-RAS-vaccinated BALB/c mice following sporozoite challenge at 2° memory time points after boosting (days 168 and 154, respectively) (**Figure 4B,D**), which remained wholly CD8 T cell-dependent (**Figure S2**). Of note, homologous *Pb*- or *Py*-RAS-boosting enriched the fraction of CS_252–260_- or CS_280–288_-specific CD8 T cells (to ∼30% and 15%, respectively) within the CD8α^lo^CD11a^hi^ compartment compared to single immunized BALB/c mice (**Figure 1B** **and Figure S3**). Importantly, these secondary CS_252–260_- or CS_280–288_-specific memory CD8 T cells are also found exclusively in the CD8α^lo^CD11a^hi^ compartment (**Figure S3**). Thus, homologous *Pb*-RAS or *Py*-RAS boosting of B6 or BALB/c mice, respectively, doubles the frequency of circulating RAS-specific 2° memory CD8 T cells and affords CD8 T cell-dependent sterilizing immunity against a stringent sporozoite challenge. Moreover, enrichment of the CS-specific responses in RAS-boosted BALB/c mice suggests that although ∼85–95% of the total initial CD8 T cell response targets antigens of undefined specificity, the CS-specific response in BALB/c mice dominates the recall response. These data for the first time reveal the effect of homologous RAS boosting on bona fide memory CD8 T cell responses, and further demonstrate that antigen-specific 2° memory CD8 T cell populations are also accurately identified using the CD8α^lo^CD11a^hi^ surrogate activation marker approach.

Consistent with the results described above, homologous boosting of *Py*-RAS immune B6 mice also doubled (on average) the frequency of RAS-induced 2° memory CD8 T cells (**Figure 4E,F**). However, these mice exhibited only modest (40%) protection against sporozoite challenge at a 2° memory time point (day 154) (**Figure 4F**). Interestingly, a second booster immunization with *Py*-RAS resulted in a sustained increase in the frequency of CD8α^lo^CD11a^hi^ T cells, which now represented on average ∼40% of the CD8 T cell compartment of the PBL at day 215 (**Figure 4E,F**). However, even this extreme commitment of *Py*-RAS-induced tertiary (3°) memory CD8 T cells did not improve protection when these mice were challenged at a 3° memory time point (day 215) (**Figure 4F**). Thus, we could not achieve substantial levels (>70–80%) of protection against *Py* sporozoite challenge in B6 mice boosted every 60–70 days and challenged 60 days after the last boost. This contrasts sharply with reports that examine protective immunity following short interval boosting (every 2–3 weeks) followed by challenge ∼14 days after the last boost [11], [29]. One clear difference between these two immunization regimens is the substantial role for CD4 T cells in protection after short-interval boost and challenge approaches in B6 mice [11], [29], whereas we could detect no role for CD4 T cells in protection against *Pb* sporozoite challenge of B6 mice, or against *Py* challenge of BALB/c mice in our long-interval prime-boost approach (**Figure S2**). These disparate results strongly suggest that the timing of RAS-immunization and sporozoite challenge significantly influences both the composition and protective capacity of the RAS-induced cellular response. Indeed, we are currently evaluating quantitative and qualitative characteristics of the total CD8 T cell response and protection following short-interval, prime-boost RAS vaccination and challenge, as well as evaluating surrogate activation marker approaches to specifically identify antigen-experienced CD4 T cells.

### Longitudinal analyses of RAS vaccine-induced CD8 T cell responses within individuals of an outbred population

**Figure 5:**
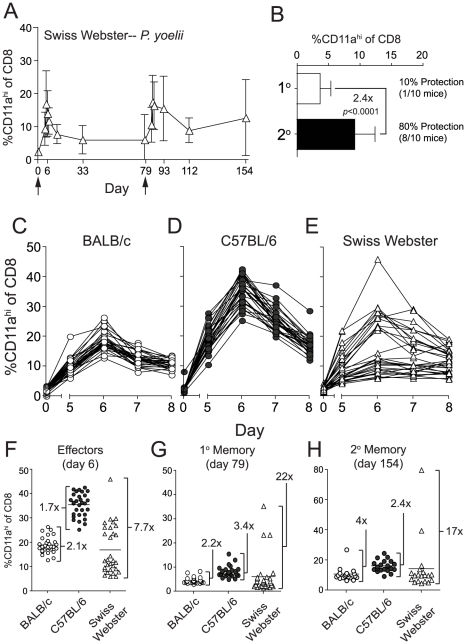
*Py*-RAS-specific CD8 T cell responses and protective immunity are markedly enhanced following prime-boost vaccination of an outbred mouse population. (**A**) The kinetics and magnitude of CD8 T cell responses in the blood of individual Swiss Webster mice following priming and boosting (arrows) with 2×10^4^ *Py*-RAS. (**B**) Fold expansion of *Py*-RAS-induced primary (1°, d79) and secondary (2°, d154) memory CD8 T cell responses in the blood of Swiss Webster mice. Numbers to the right indicate the fraction of 1° or 2° memory Swiss Webster mice that were protected following *Py* sporozoite challenge (no. protected/no. challenged×100). One hundred percent (10/10) of age-matched, naïve Swiss Webster mice challenged in parallel were parasitized. Data in **B** are mean±S.D. from 20 mice. Statistics were determined by unpaired, two-tailed *t*-tests. BALB/c (**C**), C57BL/6 (**D**) and Swiss Webster mice (**E**) (N = 30 each) were vaccinated with 2×10^4^ *Py*-RAS. Peripheral blood was collected before (day 0) and on days 5–8 following vaccination. CD8α^lo^CD11a^hi^ T cell responses were measured in individual BALB/c, C57BL/6 and Swiss Webster mice at the initial effector stage (**F**), primary memory (**G**) or secondary memory (**H**) following priming and homologous boost with 2×10^4^ *Py*-RAS as in **A**. Each symbol represents an individual mouse. Numbers in **F-H** refer to the fold difference between the highest and lowest responders within each mouse strain at each time point.

We show that BALB/c and B6 mice fall on opposite ends of the spectrum regarding their ability to resist sporozoite challenges at memory time points following either *Pb*- or *Py*-RAS long-interval prime-boost vaccination. Indeed, many studies [14], [30], [31], [32], [33], [34], [35], [36], [37] employ BALB/c mice to evaluate whole-attenuated parasite vaccine-induced protective immunity, and it is unclear how these data model CD8 T cell responsiveness and protective immunity in outbred populations, such as humans, following RAS-vaccination. Thus, we next turned our attention toward analyses of the CD8 T cell response in outbred Swiss Webster mice. Due to the lack of information on MHC alleles and antigens in outbred populations, this analysis was only made possible through development of the CD8α^lo^CD11a^hi^ surrogate activation marker approach [18]. On the population level (N = 30 mice), the kinetics and magnitude of *Py*-RAS-induced CD8 T cell responses of outbred mice mirrored those observed in inbred mice (**Figure 5A**). However, and in striking contrast to the inbred mice, the initial CD8 T cell response following *Py*-RAS-vaccination in outbred mice was not uniform and varied widely, both in magnitude and day of the peak (**Figure 5C–F**). Consistent with this, outbred mice also exhibited more variability in the magnitude of the 1° memory (**Figure 5G**) and 2° memory (**Figure 5H**) CD8 T cell response, compared to inbred mice. Similar to what was observed in both BALB/c and B6 mice singly vaccinated with *Py*-RAS, Swiss Webster mice challenged with *Py* sporozoites at a 1° memory time point (day 79) were not efficiently protected (**Figure 5B**). However, boosting Swiss Webster mice with *Py*-RAS resulted in a doubling (on average) of sporozoite-specific 2° memory CD8 T cells, and 80% of these mice were protected against a sporozoite challenge on day 154 (**Figure 5B**). Thus, CD8α^lo^CD11a^hi^ surrogate markers can be used to identify and longitudinally track protective CD8 T cell responses in outbred mice following RAS prime-boost vaccination. In addition, these data show that despite the variability in magnitude of initial RAS-induced CD8 T cell response of outbred hosts, homologous boosting increases the secondary memory CD8 T cell population and protective immunity against sporozoite challenge.

### An effector memory (T_EM_) CD8 T cell phenotype, but not anti-sporozoite antibody titers, correlates with protective immunity following *Py*-RAS vaccination of inbred and outbred mice

While protection of RAS-vaccinated mice using the long-interval (>60 days), prime-boost scenario described above is CD8 T cell dependent (**Figure S2**), RAS-immunization also elicits a strong sporozoite-specific antibody response [29], [38]. To determine whether differences in the *Py*-RAS-induced sporozoite-specific antibody response correlated with relative resistance or susceptibility to *Py* sporozoite challenge, we analyzed serum from individual BALB/c, B6 and Swiss Webster mice for sporozoite-specific IgG titers at each memory time-point. Importantly, an examination of anti-sporozoite titers at the secondary memory time point (day 154), where significant protection was achieved in BALB/c and Swiss Webster but not B6 mice (**Figures 4D** **and** **5B** **, respectively**), revealed no clear correlation between IgG titers and protection from sporozoite challenge (**Figure S4A**). Moreover, antibody titer was not significantly different between 2° memory BALB/c mice (100% protected) and 3° memory B6 mice (20% protected) (*P* = 0.0789, **Figure S4B**), or between individual protected and non-protected B6 mice (*P* = 0.4484, **Figure S4C**). Thus, reduced anti-sporozoite IgG antibody titers do not appear to explain the enhanced susceptibility of RAS-vaccinated B6 mice to sporozoite challenge.

**Figure 6:**
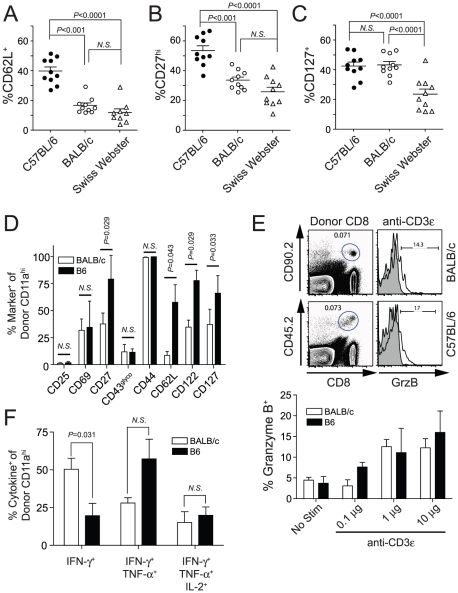
Protection correlates with an effector memory (T_EM_) phenotype on circulating *Py*-RAS-induced secondary memory CD8 T cells in inbred and outbred mice. (**A–C**) C57BL/6, BALB/c and Swiss Webster mice (N = 10/strain) were vaccinated with 2×10^4^ *Py*-RAS. Seventy-nine days later, mice received a homologous boost of 2×10^4^ *Py*-RAS. Seventy-five days after boost (day 154), circulating T cells were stained for CD8α, CD11a, CD62L, CD27 and CD127. (**D–F**) *Py*-RAS-specific, secondary memory BALB/c and C57BL/6 CD8 T cells were generated following adoptive transfer as described in Materials and Methods. (**D**) Donor-derived cells were analyzed directly *ex vivo* for surface expression of the indicated activation- or survival-associated marker. (**E**) Cells were stimulated *ex vivo* for 5 hrs using titrations of plate-bound anti-CD3ε and subsequently analyzed for the intracellular expression of granzyme B. Representative dot plots and histograms (top) and summary graph (bottom) are shown. (**F**) Cells were stimulated with 1µg/mL plate-bound anti-CD3ε for 5.5 hrs prior to staining for intracellular co-expression of IFN-γ, TNF-α and IL-2. In **A–D**, data represent the fraction of CD8α^lo^CD11a^hi^ T cells within each group expressing the indicated marker. Statistics were determined by unpaired, two-tailed *t*-tests. Data in **D–F** are mean±S.D. and represent analyses from 5 individual BALB/c and B6 recipient mice.

We next examined potential qualitative differences in phenotype and specific functional attributes of protective and non-protective memory CD8 T cells. The nature of our longitudinal analyses precluded the collection of large quantities of blood from individual immunized mice. Thus, the small clinical sample limited our initial analyses to a key subset of markers that distinguish “central memory” (T_CM_; CD62L^hi^, CD27^hi^) from “effector memory” (T_EM_; CD62L^lo^, CD27^lo^) CD8 T cell populations [39], [40], and a marker associated with memory CD8 T cell survival (IL7 receptor α chain, CD127) [41]. At 60 days post-immunization most RAS-induced memory CD8 T cells in each mouse strain, vaccinated with either *Pb*- or *Py*-RAS, expressed a T_EM_ phenotype (CD62L^lo^, CD27^lo^, CD127^lo^, data not shown). However, to more directly address potential relationships between RAS-induced memory CD8 T cell phenotype and protection we evaluated expression of the same markers after *Py*-RAS boosting of BALB/c and Swiss Webster mice (both protected) and B6 mice (not protected). The most striking difference between non-protective 2° memory CD8 T cells in B6 mice and protective memory CD8 T cells in BALB/c and Swiss Webster mice was the differential expression of CD62L (**Figure 6A**). Forty percent (on average) of *Py*-RAS-induced 2° memory cells in B6 mice expressed the CD62L^hi^ T_CM_ phenotype (**Figure 6A**). In contrast, representation of the CD62L^hi^ T_CM_ phenotype among *Py*-RAS-induced 2° memory cells in BALB/c and Swiss Webster mice was reduced 3-fold or 4-fold, respectively (**Figure 6A**). A similar trend was observed for the CD27^hi^ phenotype (**Figure 6B**), while no correlation between CD127 expression and protective capacity was observed (**Figure 6C**). Thus, non-protective 2° memory CD8 T cells in *Py*-RAS boosted B6 mice exhibit a more T_CM_ phenotype, expressing significantly higher levels of CD62L and CD27 relative to protective 2° memory CD8 T cells in BALB/c or Swiss Webster mice (**Figure 6A,B**).

As a complimentary approach, we next performed a series of adoptive transfer studies in order to more clearly identify and directly compare RAS-induced 2° memory CD8 T cells in BALB/c and B6 mice. We transferred 8×10^4^ *Py*-RAS-induced, CD8α^lo^CD11a^hi^ 1° memory (d78) CD8 T cells into allelically disparate BALB/c and B6 recipients, which were subsequently immunized with *Py*-RAS to generate populations of endogenous 1° memory and allelically marked 2° memory parasite-specific CD8 T cells. One month after the booster immunization, we performed extensive phenotypic and functional analyses of the donor-derived, 2° memory CD8 T cells in each B6 and BALB/c recipient mouse. Surface expression of many markers, such as CD25, CD69, CD43^glyco^ (**Figure 6D** and **Figure S5**) were indistinguishable between these populations. In addition, we found no statistically significant differences in expression of integrins (β_1_, β_2_, β_7_, α_M_, α_X_, α_E_, α_4_, α_5_ or α_6_) or inhibitory receptors (PD-1, LAG-3, 2B4, CD160, KLRG-1 or CTLA-4) on RAS-induced, 2° memory CD8 T cells in B6 and BALB/c mice (data not shown). However, in line with our initial observations, RAS-induced, non-protective 2° memory CD8 T cells in B6 mice exhibit a more T_CM_-like phenotype, relative to BALB/c mice, characterized by relatively higher proportions of CD27 and CD62L expressing cells (**Figure 6D** **and Figure S5**). We also observed significantly higher CD122 and CD127 expression on RAS-induced, 2° memory CD8 T cells in B6 mice (**Figure 6D** **and Figure S5**). Collectively, our phenotypic analyses support the notion that a T_EM_ phenotype among RAS-induced, parasite-specific CD8 T cells strongly correlates with protection against liver stage *Plasmodium* infection.

To examine specific functional attributes of RAS-induced memory CD8 T cells in BALB/c and B6 mice we relied on polyclonal TCR cross-linking to trigger the *ex vivo* induction of Granzyme B and inflammatory cytokine expression by allelically marked, 2° memory CD8 T cells. We found that similar fractions of BALB/c and B6 2° memory CD8 T cells expressed Granzyme B in response to dose-titrations of plate-bound anti-CD3ε (**Figure 6E**). Moreover, we observed equivalent IFN-γ production (% positive and MFI) by RAS-induced CD8α^lo^CD11a^hi^ 2° memory CD8 T cells in BALB/c and B6 mice (data not shown). Interestingly, however, a significantly higher fraction of B6 2° memory CD8 T cells co-expressed TNF-α and IFN-γ relative to BALB/c 2° memory CD8 T cells, the majority of which expressed IFN-γ alone (**Figure 6G**). Collectively, these data show that a T_EM_ phenotype, but neither Granzyme B nor polyfunctional cytokine expression, correlates with protective anti-*Plasmodial* liver stage immunity mediated by RAS-induced memory CD8 T cells.

### Extreme numerical requirements for CD8 T cell-mediated protective immunity to sporozoite challenge

**Figure 7:**
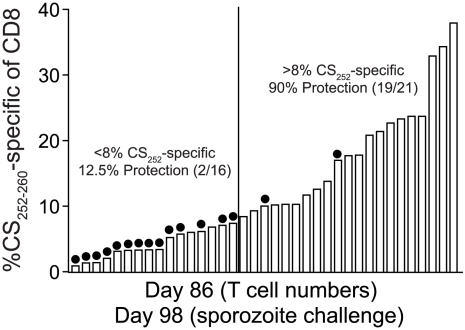
Sterilizing anti-*P. berghei* sporozoite immunity in BALB/c mice is associated with memory CD8 T cell responses of single antigenic-specificity that exceed 8% of all circulating CD8 T cells. BALB/c mice were DC-CS_252–260_ prime, LM-CS_252–260_ boost immunized as previously described [16]. Eighty-six days following immunization, peripheral blood from individual mice was assayed for the frequency of CS_252–260_-specific memory CD8 T cells using intracellular IFN-γ cytokine staining. Mice are ranked according to the magnitude of the CS_252–260_-specific memory CD8 T cell response. Three days after T cell analyses, mice were challenged with 1000 *Pb* sporozoites and blood stage parasitemia was evaluated in individual mice with Giemsa stain. Non-protected mice are indicated with filled circles. Numbers above the graph refer to percent protection among mice scoring above or below the 8% circulating CS_252–260_-specific memory CD8 T cell threshold, which is based on the clearest visual break-point between non-protected and protected mice each ranked according to the magnitude of the CD8 T cell response. Ninety-one percent (20/22) of age-matched, naïve BALB/c mice challenged in parallel were parasitized.

We previously reported that the numerical threshold for protection of BALB/c mice against *Pb*-sporozoite challenge mediated solely by memory CS_252–260_-specific CD8 T cells is exceedingly high (>1% of PBL (refs [16], [42]), or >8% of CD8 T cells, **Figure 7**). One explanation for the enormously high threshold for sterilizing protection in that scenario is that protective memory CD8 T cells recognize only a single antigenic determinant from *P. berghei*. On the other hand, RAS-vaccination has been shown to elicit protective CD8 T cells targeting non-CS antigens [14], [15] and our data are consistent with the majority of RAS-induced CD8 T cells targeting non-CS antigens (**Figure 1B** **and Figure S3**). Thus, broadening the number of antigens (i.e. additional parasite-derived proteins that may be more efficiently processed or presented compared to CS) through whole parasite RAS-vaccination may lower the numerical requirements for protective immunity mediated by memory CD8 T cells. However, when we tabulated memory CD8 T cell responses in groups of RAS-vaccinated inbred and outbred mice that resisted sporozoite challenge, we identified similarly extreme numerical relationships between circulating memory CD8 T cell responses and protective anti-sporozoite immunity (**Table 1**). This is most evident for RAS-induced protective immunity against *P. yoelii*, which replicates faster in the liver [43] and exhibits a lower ID_50_ [32] compared to *P. berghei*, and thus, may better mimic the virulence of *P. falciparum* in humans. For example, anti-*Py* memory CD8 T cell responses representing ∼9% of CD8 T cells in BALB/c mice and ∼19% of CD8 T cells in Swiss Webster mice confer protection against a *Py*-sporozoite challenge, and responses exceeding 40% of CD8 T cells failed to efficiently protect B6 mice. Thus, the numerical requirements for sterilizing immunity following *Plasmodium* RAS-vaccination are extraordinarily high, regardless of whether the protective pool of memory CD8 T cells react to a single antigenic determinant after subunit vaccination, or whether the CD8 T cell response is directed against a broader set of antigenic determinants after whole-parasite immunization.

**Table 1:** Extreme commitment of the CD8 T cell compartment is associated with protection against *Plasmodium* sporozoite challenge.

	BALB/c	C57BL/6	Swiss Webster
*P. berghei*	4.2±0.53%^a^^,^ b (n = 25)^c^	11±1.0% (n = 15)^d^	12±2.8% (n = 23)^e^
*P. yoelii*	8.8±1.2% (n = 15)^d^	N.I.^f^	19±4.5% (n = 17)^e^

a Numbers are percent (±S.E.M.) of circulating CD8 T cells that exhibit an antigen-experienced phenotype (CD8α^lo^CD11a^hi^) in RAS-vaccinated mice that resisted homologous challenge with 1000 *P. berghei* or *P. yoelii* sporozoites.

b RAS-induced responses were calculated after subtracting the CD8α^lo^CD11a^hi^ responses of individual naïve mice measured prior to RAS-immunization.

c Single-vaccinated mice (1 dose of 2×10^4^ *Pb*- or *Py*-RAS).

d Double-vaccinated mice (2 doses of 2×10^4^ *Pb*- or *Py*-RAS).

e Double and triple-vaccinated mice (2 or 3 doses of 2×10^4^ *Pb*- or *Py*-RAS).

f N.I. = Not Included. Only groups of mice exhibiting >80% protection after 1, 2 or 3 RAS-vaccinations were included in this analysis.

## Discussion

Although a critical protective role for CD8 T cells in RAS-immune mice was established more than 25 years ago, the characteristics of the protective CD8 T cell response remained essentially undefined due to the lack of defined *Plasmodium* epitopes. Here, we used surrogate activation markers to identify and longitudinally track RAS-induced CD8 T cell populations in the blood of individual hosts. This approach enabled us to describe specific quantitative and qualitative characteristics of memory CD8 T cell populations that mediate protection against sporozoite challenge. Moreover, the surrogate activation marker approach allowed us to monitor RAS vaccine-induced CD8 T cell responses in individuals within outbred populations of mice, without *a priori* knowledge of MHC alleles or parasite-specific antigenic determinants. These latter analyses revealed that, despite variability to the initial immunization, prime-boost RAS-vaccination effectively enhances parasite-specific memory CD8 T cell responses and affords sterilizing protective immunity among individuals of an outbred population. Collectively our studies show that independent of genetic background, extremely high frequencies of RAS-induced memory CD8 T cells are required, but may not always suffice for sterilizing anti-*Plasmodial* immunity, information directly relevant to ongoing efforts to translate attenuated whole-malaria parasite vaccines to humans.

We previously reported that an extraordinarily large frequency of circulating CS-specific memory CD8 T cells generated by subunit vaccination is required to protect BALB/c mice against a stringent *Pb* sporozoite challenge [16]. In that study, memory CD8 T cell populations were generated such that they only targeted a single antigenic determinant derived from the parasite CS protein, CS_252–260_. Herein we report the striking observation that diversifying the targets of the CD8 T cell response through whole-attenuated-parasite vaccination only minimally (if at all) reduces the numerical requirements for memory CD8 T cell-mediated protective immunity. For example, we show that following *Pb*-RAS vaccination of BALB/c mice (the scenario in which protection is easiest to achieve) resistance to sporozoite challenge at a memory time point is associated with ≥4% of the CD8 T cell compartment exhibiting the antigen-experienced phenotype (CD8α^lo^CD11a^hi^). Thus, the magnitude of the RAS-induced, poly-specific memory CD8 T cell response associated with protection against *P. berghei* challenge is only ∼2-fold lower than the mono- (CS_252–260_)-specific memory CD8 T cell response (∼8% of the CD8 T cell compartment). Additionally, protection against *Pb* is associated with even larger memory CD8 T cell responses in B6 and outbred Swiss Webster mice (11% and 12%, respectively). Further, resistance to *P. yoelii* has even more extreme requirements, with protection associated with RAS-induced memory CD8 T cell responses exceeding ∼9 or ∼19% of the CD8 T cell compartment in BALB/c and Swiss Webster mice, respectively. Thus, our data demonstrate that poly-specific memory CD8 T cell-mediated sterilizing immunity to sporozoite challenge, regardless of the relative virulence of the *Plasmodium* species, requires commitment of a substantial fraction of the entire CD8 T cell compartment.

This extreme numerical requirement is perhaps not surprising given the extraordinarily low ratio of *Plasmodium*-infected cells to total hepatocytes in the mammalian host following challenge with physiological numbers of sporozoites (∼1000). Indeed, the gold standard readout for protection against sporozoite challenge is sterilizing immunity, or the prevention of blood stage infection. From a conservative perspective, this level of protection requires that each of a maximum of 1000 infected hepatocytes (among >10^8^ or >10^11^ total hepatocytes in the mouse or human liver, respectively) be targeted through direct CTL activity or indirectly via the release of cytokines by parasite-specific memory CD8 T cells in order to prevent the development of blood stage infection. Thus, each RAS-induced memory CD8 T cell must surveil an extremely large number of hepatocytes in order to identify all cells that harbor parasites. The exceedingly low number of infected cells among the whole liver (needle in the haystack [16]), coupled with the fact that every single infected cell must be successfully targeted to prevent blood stage infection, is the likely explanation for why the numerical requirements for memory CD8 T cell-mediated, anti-*Plasmodial* liver stage immunity are so high.

While it is unclear why commitment of nearly 40% of the CD8 T cell compartment to the anti-*Plasmodial* memory CD8 T cell response is insufficient to effectively protect B6 mice, our studies extend the literature [11], [20] by showing that host genetics play a significant role in determining the outcome of sporozoite challenge following RAS-vaccination at bona fide memory time points. We were unable to detect CS-specific CD8 T cell responses in RAS-vaccinated B6 mice (data not shown), which could account for reduced protection. However, experiments have consistently shown that RAS-immune B10.D2 mice are equally as difficult to protect as B6 and B10 mice [11], [20]. Importantly, B10.D2 mice express the same MHC genes as BALB/c mice and thus are able to mount CD8 T cell responses against the defined CS epitopes. Thus, vaccine-induced CD8 T cell responses against the defined immunodominant CS determinant are not sufficient for protection, underscoring the role of non-MHC-linked genes in regulating RAS-induced, anti-liver stage immunity.

Another hypothesis to explain the dramatic susceptibility of hyper-*Py*-RAS-immune B6 mice is that critical phenotypic or functional attributes of the memory CD8 T cell response, that differ in B6 and BALB/c, regulate protective liver stage immunity. Although we found no differences in granzyme B, IFN-γ, TNF-α or IL-2 production by BALB/c- compared to B6-derived, RAS-induced memory CD8 T cells we did observe differential expression of key molecules that differentiate T_CM_ from T_EM_ populations. RAS-induced memory CD8 T cell populations in B6 mice consistently exhibited elevated proportions of CD62L^hi^CD27^hi^ populations (T_CM_ phenotype) compared to the predominantly T_EM_ populations found in BALB/c and Swiss Webster mice. These data demonstrate a clear correlation between the expression of the T_EM_ (CD27^lo^CD62L^lo^) phenotype of secondary memory CD8 T cells and the ability of RAS-immune BALB/c and Swiss Webster mice to resist sporozoite challenge. The reason(s) for the difference in memory phenotype between RAS-immune B6 and BALB/c or/Swiss Webster mice are unknown. Interestingly, a recent report using a sensitive *in vivo* assay suggests that CS antigen persists for long periods of time after RAS immunization [44]. These studies were carried out only in BALB/c mice due to reagent availability. Given the potential of prolonged antigen encounter to influence memory T cell phenotype, it will be of interest to determine if antigen fails to persist in B6 mice and accounts for the altered T cell phenotype and reduced protection after RAS-immunization. Finally, it should be noted that, due to the enormous numbers of memory CD8 T cells required for sterilizing immunity, adoptive transfer studies to compare the per cell protective capacity of CD8α^lo^CD11a^hi^ memory populations from RAS-immune BALB/c and B6 have not succeeded Although it may be possible to measure reductions in parasite liver burden in CD8 T cell-recipient mice using quantitative PCR, this generally requires challenging mice with supraphysiological doses of sporozoites, a scenario that we wished to avoid. In addition, our interests are focused on the properties of memory CD8 T cells that result in sterilizing immunity and it is currently unclear how reduction in parasite burden after high dose challenge models complete elimination of infected hepatocytes. Clearly many other characteristics of the RAS-induced memory CD8 T cell response may contribute to protective immunity and warrant further investigation. Importantly, our surrogate activation marker approach should allow for detailed, prospective characterization of RAS-induced memory CD8 T cell responses in individual hosts, so that potential links between specific memory CD8 T cell attributes and protection can be evaluated. The identification of additional factors that correlate with or determine CD8 T cell-mediated protective immunity following RAS-vaccination should provide key insight into the pathways of protective CD8 T cell-mediated immunity elicited through whole-parasite vaccination.

Our data highlight the utility of the surrogate activation marker approach for understanding *Plasmodium*-induced CD8 T cell responses in genetically diverse populations by providing a framework with which the field can begin to address several additional critical knowledge gaps. First, use of outbred rodents to evaluate vaccine-induced responsiveness and protective immunity is much more likely to mimic responses in genetically diverse humans or non-human primates. Identifying and characterizing individual-to-individual variability in response to vaccination should provide additional critical information that will complement data obtained through studying the highly overlapping responses in genetically identical, inbred rodent populations. Second, our studies provide a framework with which to optimize whole parasite immunization. Identifying ways to enhance potentially suboptimal delivery routes, vaccine doses or schedules, based on a quantitative and qualitative assessment of the total CD8 T cell response, will significantly improve efforts to optimize RAS-vaccination, or any other candidate vaccine delivery approaches. Lastly, the surrogate activation marker approach now permits direct comparisons between RAS and the genetically attenuated parasite (GAP) vaccines. Recent work has shown that such genetically attenuated *Plasmodium* parasites, harboring defined mutations in one or more key genes required for full liver stage differentiation, afford CD8 T cell-dependent protective immunity in rodents [33], [45]. Moreover, it has been shown that the targeted gene(s) precisely control the point during liver stage development that the GAP arrests [31], [45], [46], [47]. Whether early arresting or late arresting GAP-vaccine candidates differentially impact the protective characteristics of the CD8 T cell response is unknown. Given the potential safety advantage of GAP vaccination, these will be critically important questions that can now be directly addressed using surrogate activation markers to identify vaccination-induced effector and memory CD8 T cell responses.

## Materials and Methods

### Ethics statement

All animal studies and procedures were approved by the University of Iowa Animal Care and Use Committee, under PHS assurance, Office of Laboratory Animal Welfare guidelines.

### Mice

Specific pathogen-free BALB/c, C57BL/6, and Swiss Webster mice were purchased from the National Cancer Institute (NCI) and housed at the University of Iowa animal care unit at the appropriate biosafety level.

### Parasites

Female *Anopheles stephensi* mosquitoes infected with either *P. berghei* (NK65) or *P. yoelii* (17XNL) were purchased from the New York University insectary.

### Immunizations

*P. berghei* and *P. yoelii* sporozoites were isolated from the salivary glands of infected *A. stephensi* mosquitoes. Sporozoites were attenuated by exposure to 200 Gy (20,000 rads). Mice were immunized with 200 to 100,000 RAS i.v. Boosted mice received 20,000 RAS no less than 60 days apart. In some experiments mice were injected with 60 mg/kg primaquine (Sigma-Aldrich, St. Louis, MO) i.p. on days 5 and 6 following RAS immunization. Subunit immunizations were performed as previously described [16]. Briefly, BALB/c mice were primed via tail vein injection of 1×10^6^ splenic dendritic cells coated with peptides corresponding to CS_252–260_ of *P. berghei* (DC-CS_252–260_). Seven days later, mice were boosted with 2×10^7^ CFU of recombinant *Listeria monocytogenes* expressing the CS_252–260_ determinant as a secreted minigene (LM-CS_252–260_).

### Sporozoite challenges

*P. berghei* and *P. yoelii* sporozoites were isolated from the salivary glands of infected *A. stephensi* mosquitoes. Immunized and naïve age-matched mice were challenged with 1000 sporozoites i.v. Thin blood smears were performed 10 days after sporozoite challenge. Parasitized red blood cells were identified by Giemsa stain and oil-immersion (1000×) light microscopy. Protection is defined as the absence of blood stage parasites. At least 10 fields (∼10–15,000 red blood cells) were examined for each mouse designated as protected. Protected mice were subsequently rechallenged following T cell depletion to verify that protection was CD8 T cell-dependent.

### Quantification and characterization of antigen-specific CD8 T cells

RAS vaccine-induced CD8 T cell populations were identified by staining spleen or liver single cell suspensions, or peripheral blood following lysis of red blood cells, with anti-CD8α clone 53–6.7 (eBioscence, San Diego, CA) and anti-CD11a (LFA-1α) clone M17/4 (eBioscience) antibodies. Sporozoite-specific CD8 T cells were phenotyped by staining cells with anti-CD27 clone LG.7F9, anti-CD43 clone 1B11, anti CD62L clone MEL-14, anti-CD127 clone A7R34, anti-CD25 clone PC61, anti-CD69 clone H1.2F3, anti-CD44 clone IM7, anti-CD122 clone 5H4 antibodies, all from eBioscience. In some experiments, 8×10^4^ *Py*-RAS-induced primary memory cells from CD90.2^+^ BALB/c or CD45.2^+^ B6 mice were adoptively transferred to naïve, congenic (CD90.1^+^ or CD45.1^+^) recipients. One day following transfer, recipients were boosted with 1×10^5^ *Py*-RAS. Thirty-three days later, splenocytes were stimulated *ex vivo* in anti-CD3ε-coated wells. BALB/c and B6 donor cells were identified by CD11a^hi^CD8α^lo^CD90.2^+^ or CD11a^hi^CD8α^lo^CD45.2^+^ surface staining and further characterized by intracellular staining for IFN-γ, TNF-α and IL-2, or Granzyme B. CS_252–260_- and CS_280–288_-specific CD8 T cells were identified by incubating peripheral blood leukocytes with K^d^/CS_252–260_-APC labeled tetramers or K^d^/CS_280–288_-APC labeled tetramers, respectively. Cells were then stained with anti-CD8α, anti-CD90.2 and anti-CD11a. Following subunit immunization, the frequency of circulating CS_252–260_-specific CD8 T cells was determined by *ex vivo* intracellular cytokine staining for IFN-γ following a 5.5 hour incubation with brefeldin A in the presence or absence of CS_252–260_ peptide-coated P815 cells were used as antigen presenting cells. Cells were analyzed using a BD FACSCanto and data was analyzed using FLOWJO Software (Tree Star, Inc, Ashland, OR). All animals were pre-bled prior to RAS vaccination to establish individual background circulating CD8α^lo^CD11a^hi^ T cell frequencies.

### T cell depletions

Immunized mice were injected with 0.4 mg i.p. rat IgG, anti-CD4 (clone GK1.5), or anti-CD8 (clone 2.43) antibodies on day −3 and day −1 prior to challenge with sporozoites. Depletion was verified by analyzing CD4 (clone RM4-5) and CD8 (clone 53-6.7) T cell populations in the blood of individual mice prior to challenge. In each case, the relevant population represented <0.5% of the PBL.

### Sporozoite-specific antibody titer

The serum sporozoite-specific antibody titer from immunized mice was determined by the indirect fluorescent antibody test (IFAT). Sporozoites were air dried on a multiwell microscope slide (Cel-Line Thermo Scientific) and blocked with 1% BSA/PBS. Sporozoite-specific IgG antibodies were detected by incubating with Cy3-conjugated goat anti-mouse IgG (Jackson Immunoresearch Laboratories). Titers are expressed as the inverse of the lowest dilution of serum that retained immunoreactivity against air-dried sporozoites.

## Supporting Information

Figure S1 CD8α^lo^CD11a^hi^ memory cells, but not naive CD8α^hi^CD11a^lo^ cells, sorted from *Py*-RAS-immune mice specifically expand in naive recipient hosts following *Py*-RAS vaccination. Splenic memory CD8α^lo^CD11a^hi^ and naive CD8 T cells were simultaneously sorted from (CD45.2^+^) B6 donor mice 78 days following immunization with 2×10^4^ *Py*-RAS. 2×10^5^ sort-purified memory or naive cells were transferred to naive (CD45.1^+^) recipient B6 mice one day before immunization with 1×10^5^ Py-RAS. The frequency of circulating donor-derived (CD45.2^+^) CD8 T cells in each recipient mouse was monitored starting on day 4 post-immunization.
(0.44 MB EPS)

Figure S2 Depletion of CD8 T cells, but not CD4 T cells, abrogates protection in *Pb*- and *Py*-RAS vaccinated C57BL/6 and BALB/c mice, respectively. (**A**) Naïve C57BL/6 mice (N = 20) were vaccinated via tail vein with 2×10^4^ *Pb*-RAS. Eighty-six days later, mice were boosted with 2×10^4^ *Pb*-RAS. Eighty-two days following boost the frequency of CD8α^lo^CD11a^hi^ T cells in the peripheral blood was evaluated and individual mice were ranked according to the magnitude of the secondary memory CD8 T cell response. Groups of mice, as indicated, were treated with 0.4 mg rat IgG, anti-CD4 (GK1.5) or anti-CD8 (2.43) 3 days and 1 day prior to challenge with 1000 *Pb* sporozoites. Complete CD8 or CD4 T cell depletion was verified by collecting a small volume (∼20 µL) of peripheral blood from each individual mouse on the day of sporozoite challenge followed by staining for both CD4 (RM4-5) and CD8 (53-6.7) (data not shown). Numbers above the graph refer to the percent of T cell-depleted mice that remained protected (no. protected/no. challenged×100) following *Pb* sporozoite challenge. Blood stage parasitemia was evaluated by Giemsa stain. (**B**) Similar to **A**, except CD8 T cell-dependent protection was evaluated in BALB/c mice primed and boosted with 2×10^4^ *Py*-RAS. In **B**, mice were challenged with 1000 *Py* sporozoites following T cell depletions.
(0.47 MB EPS)

Figure S3 Homologous boosting with RAS enriches the representation of CS_252–260_- or CS_280–288_-specific CD8 T cells within the vaccine-induced CD8α^lo^CD11a^hi^ population. (**A**) Representative dot plots showing the fraction of circulating CD8 T cells exhibiting an antigen-experienced phenotype (CD8α^lo^CD11a^hi^) in a naïve mouse (top), memory *Pb*-RAS-immune (middle) and *Py*-RAS-immune (bottom) mouse 7 days following homologous RAS boost. (**B**) Samples from memory mice (shown in **A**) subgated into CD11a^hi^ and CD11a^lo^ populations and analyzed for K^d^/CS_252–260_ or K^d^/CS_280–288_ tetramer staining. (**C**) Fraction of CD11a^hi^ cells that are K^d^/CS_252–260_ tetramer+ following primary *Pb*-RAS vaccination (day 7) or one week following homologous *Pb*-RAS boosting of memory mice (day 61+7). (**D**) Similar to **C**, except *Py*-RAS immune mice were analyzed. For **C** and **D**, data are mean ± SD (n = 3/group). Statistics were determined by unpaired, two-tailed t-tests.
(0.81 MB EPS)

Figure S4 Anti-*P. yoelii* sporozoite serum IgG antibody titers are not significantly different between protected and non-protected mice following *Py*-RAS prime-boost vaccination. (**A**) Anti-*Py* sporozoite IgG antibody titers in the serum of individual BALB/c, C57BL/6 and Swiss Webster mice at secondary (2°) and tertiary (3°) memory time-points were evaluated. Titers form individual mice are shown. (**B**) Comparison of serum anti-sporozoite titers among protected 2° memory BALB/c and non-protected 3° C57BL/6 mice. (**C**) Anti-sporozoite titers were averaged among the 6 protected or the 14 non-protected 2° and 3° memory B6 mice shown in **Fig. 4F**. Statistics were determined by unpaired, two-tailed t-tests.
(0.49 MB EPS)

Figure S5 Cell surface phenotype of RAS-induced, secondary memory CD8 T cells from BALB/c and B6 mice. Allelically marked, secondary memory CD8 T cells in BALB/c and B6 mice were generated as described in Materials and Methods. Briefly, 8×10^4^ *Py*-RAS-induced, primary memory CD8α^lo^CD11a^hi^ T cells from CD90.2^+^ BALB/c or CD45.2^+^ B6 mice were transferred to CD90.1^+^ or CD45.1^+^ congenic recipients, respectively. One day following transfer, recipient mice were vaccinated with 1×10^5^ *Py*-RAS. Thirty-three days later, splenocytes were harvested and stained with CD8α, CD11a and CD45.2 or CD90.2 to identify B6 or BALB/c secondary memory CD8 T cells, respectively. Aliquots of cells were subsequently stained for expression the indicated CD8 T cell activation or survival marker. Representative dot plot and histograms are shown for a single BALB/c and B6 recipient mouse. Numbers indicate the fraction of donor-derived, *Py*-RAS-specific secondary memory CD8 T cells expressing the indicated marker. Isotype control antibody staining for each activation or survival marker is shown as a shaded histogram.
(1.60 MB EPS)
